# Declines in freshwater mussel density, size and productivity in the River Thames over the past half century

**DOI:** 10.1111/1365-2656.13835

**Published:** 2022-11-27

**Authors:** Isobel Ollard, David C. Aldridge

**Affiliations:** ^1^ Aquatic Ecology Group, David Attenborough Building University of Cambridge Cambridge UK

**Keywords:** biodiversity loss, biomass, freshwater mussels, growth rate, invasive species, population dynamics, unionids, zebra mussels

## Abstract

1. A pioneering, quantitative study published in Journal of Animal Ecology in 1966 on freshwater mussel populations in the River Thames, UK, continues to be cited extensively as evidence of the major contribution that mussels make to benthic biomass and ecosystem functioning in global river ecosystems.
2. Ecological alteration, as well as declines in freshwater mussel populations elsewhere, suggest that changes to mussel populations in the River Thames are likely to have occurred over the half century since this study.
3. We resurveyed the site reported in Negus (1966) and quantified the changes in mussel population density, species composition, growth patterns and productivity.
4. We found large declines in population density for all unionid species. The duck mussel *Anodonta anatina* decreased to 1.1% of 1964 density. The painter's mussel *Unio pictorum* fell to 3.2% of 1964 density. The swollen river mussel *Unio tumidus* showed statistically nonsignificant declines. In contrast to 1964, in 2020 we found no living specimens of the depressed river mussel *Pseudanodonta complanata* (classified as Vulnerable by the IUCN Red List) but found new records of the invasive, nonnative zebra mussel *Dreissena polymorpha* and Asian clam *Corbicula fluminea*. Additionally, we found strong decreases in size‐at‐age for all species, which now grow to 65–90% of maximum lengths in 1964. As a result of reduced density and size, estimated annual biomass production fell to 7.5% of 1964 levels.
5. Since mussels can be important to ecosystem functioning, providing key regulating and provisioning services, the declines we found imply substantial degradation of freshwater ecosystem services in the River Thames, one of the UK's largest rivers. Our study also highlights the importance to conservationists and ecologists of updating and validating assumptions and data about wild populations, which in the present era of anthropogenic ecosystem alteration are undergoing significant and rapid changes. Regular population surveys of key species are essential to maintain an accurate picture of ecosystem health and to guide management.

## Graphical abstract

Freshwater mussels in the River Thames have declined by over 90% and are growing more slowly, compared with a study published in *Journal of Animal Ecology* 56 years ago. Drivers may include changing nutrient levels and the arrival of invasive species. The loss of these ecosystem engineers could have crucial impacts.

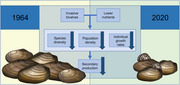

## 1 INTRODUCTION

Freshwater mussels (Unionida) are important ecosystem engineers in lakes and rivers world‐wide (Chowdhury et al., 2016; Gutiérrez et al., 2003), altering ecosystem functioning in several key ways (Vaughn, 2018; Vaughn & Hakenkamp, 2001). As filter feeders, mussels remove algae and other organic particles from the water column, transferring pelagic nutrients to the benthos (Pusch et al., 2001; Strayer, 2014; Vaughn et al., 2004) and suppressing algal blooms (Atkinson et al., 2013; Kim et al., 2011). Mussel shells provide a heterogeneous substrate which enables colonisation by epiphytic and epizootic species, and creates flow refugia for benthic invertebrates (Ilarri et al., 2018; Vaughn & Spooner, 2006). Unionids also cause sediment bioturbation, increasing oxygenation (Boeker et al., 2016). As a result, the presence of unionids in freshwater ecosystems can be associated with increased invertebrate biodiversity (Aldridge et al., 2007; but see Richter et al., 2016).

However, unionid mussels are threatened globally, with 45% of species classed as near threatened, threatened or extinct (Lopes‐Lima et al., 2018). Major drivers of decline include the impacts of invasive species including nonnative bivalves, eutrophication and pollution, habitat modification, the loss of fish that act as larval hosts, and climate change (Lopes‐Lima et al., 2017). As a result, significant declines in mussel populations have been reported (e.g. Hornbach et al., 2017; Karatayev et al., 2012; Parmalee & Polhemus, 2004; Sickel et al., 2007; Strayer & Fetterman, 1999). Historical data are crucial to improving our understanding of these declines, since it offers a baseline against which present‐day populations and habitats can be compared. This can help to identify environmental drivers of change, as well as to avoid ‘shifting baseline syndrome’ (Pauly, 1995). Typically, such data may include parameters such as population density and species composition, which provide a static snapshot of the mussel community. These can also be combined with measures of individual growth rates to calculate secondary production across the community, providing a dynamic metric that integrates both individual‐ and population‐level processes (Benke, 2010; Dolbeth et al., 2012).

One of the earliest studies to undertake a fully quantitative assessment of unionid population dynamics was conducted by Christina Negus in 1963‐4 (Negus, 1966) in the River Thames at Reading, southern England. This study established estimates for population density, individual growth rates and productivity in unionid populations, and demonstrated the significant contribution of unionids to benthic biomass. It continues to be cited as a quantitative assessment of mussel population dynamics and life‐history parameters (e.g. Benke, 2010; Czerniejewski et al., 2021; Jones & Neves, 2011; Lopes‐Lima et al., 2017; Zieritz et al., 2021). In the intervening years the Thames has undergone significant changes, including a reduction in anthropogenic nutrient input (Bowes et al., 2012; Howden et al., 2010; Neal, Jarvie, et al., 2010) and an increase in colonisations by invasive nonnative species, including the zebra mussel *Dreissena polymorpha* and the Asian clam *Corbicula fluminea* (Jackson & Grey, 2013; Keller et al., 2009). Negus' study therefore offers a valuable source of historical data to assess mussel population trajectories over the past half century in this major UK waterway.

Revisiting the site first surveyed by Negus in 1964 offers the opportunity to add to a developing picture of mussel decline across Europe (e.g. Arter, 1989; Lewandowski & Kołodziejczyk, 2014; Ożgo et al., 2021; Timm et al., 2006). Significantly, these data also allow us to assess changes in individual growth rates, leading to changing secondary production. We resampled unionid populations in the River Thames at Reading following sampling procedures used in 1964. We compared species composition, population density and species‐specific growth rates, as well as estimates of whole‐reach biomass and annual productivity, to assess the changes in unionid populations over the intervening 56 years.

## 2 MATERIALS AND METHODS

### 2.1 Study area

Sampling was conducted on 22 September 2020 in the River Thames adjacent to Wokingham Waterside Centre (51°27′35.7″N 0°56′34.3″W). The Thames is a 346 km river with a densely populated catchment covering the southeast of England, comprising both tidal and nontidal stretches and with 45 navigation locks and associated weirs. The study area was located in the nontidal stretch, approximately 152 km downstream of the source, and directly downstream of the city of Reading. Mean flow for the study area is 37.9 m^3^ s^−1^ (data from the UK National River Flow Archive). By consulting maps published in Mann (1965) and Negus (1966) we were able to resurvey the identical locality to that surveyed in 1964, a 250 m stretch of river varying in width from 50 to 65 m.

### 2.2 Data collection

Sampling was designed to replicate as closely as possible the methods employed by Negus (1966). Consultation of the original paper was supplemented by in‐person discussions with the original author. We sampled across four depth zones: 0–1 m (*n* = 32), 1–2 m (*n* = 32), 2–3 m (*n* = 14) and 3–4 m (*n* = 15), for a total of 93 samples, compared with a total of 24 samples conducted by Negus. We took equal numbers of replicates from each side of the river for each depth zone and allocating sampling effort proportionally to the different microhabitats present. Sampling in the 0–1 m and 1–2 m depth zones was conducted using randomly placed 1m^2^ quadrats, with all live unionids within the quadrat area collected and transported to the laboratory for measurement. Sampling in the 2–3 m and 3–4 m zones was conducted by dredging (dredge width 45 cm, mesh size 15 mm) from a boat along replicate 20 m‐long upriver transects. Since dredge transects covered a greater area than the quadrats in shallower zones, we conducted fewer replicates for these deeper zones. The dredges used in 1964 and the present study were similar in design and conformed to the National Rivers Authority (1996) and Environment Agency (present) specifications, including a rectangular frame and angled blade to enable sampling from sediments including gravel, silt and mud. The dredges were also operated in a similar way, with samples collected by towing from a motor boat. The total area dredged was 580 m^2^, compared with a total dredged sample area of 23.22 m^2^ in 1964.

All live mussels and all unionid shells were identified to species and recorded. For live unionids, we measured the length (longest anterior‐to‐posterior axis), height (dorsal–ventral axis) and width (left–right axis) using digital callipers. We additionally measured the length of each shell annulus (along its longest anterior‐to‐posterior axis). These are distinctive dark bands on the shell formed during periods of temporary growth cessation and have been confirmed to be annual (Rypel et al., 2008), including for populations in the Thames (Negus, 1966). They can therefore be used as a reliable measure of a mussel's yearly growth (Aldridge, 1999).

We dissected a subset of 50 mussels, distributed across species, sampling depths and sizes, to measure shell wet mass and total wet mass separately in order to calculate an estimate of biomass production, following the method reported by Negus. To limit the extent of destructive sampling, we regressed wet mass on length and used this to interpolate total and shell wet mass for the remaining individuals. We report these equations for future reference in Table S1.

Data for mussel populations in 1964 were obtained from Negus (1966) and extracted from graphs using the software DataThief III (Tummers, 2006). Data used for comparison were those reported from ‘1964, unheated’ surveys. Additional surveys reported from 1963 and from heated effluents near the now‐closed Earley Power Station offered less complete and less comparable data and were excluded.

Water quality monitoring data were obtained from the Environment Agency for the River Thames at Caversham Weir monitoring point (sampling point ID: TH‐PTHR0080), approximately 2 km upstream of our sampling location. Data from 2000 to present are publicly available (Environment Agency, 2021) and data for 1972–1999 were obtained via a Freedom of Information request.

This study did not require ethical approval and no licences were required for the collection of mussels.

### 2.3 Data analysis

Standard statistical tests were conducted in R v3.6.2, using core functions (R Core Team, 2019). We compared population densities, age structure, growth rates, biomass and annual production between 1964 and 2020 samples. Comparative analyses with 1964 data were constrained by the lack of raw data for 1964, with only selected summary statistics available; this was generally restricted to mean values, with measures of spread not reported. As a result, we were unable to conduct standard statistical comparisons for some of the analyses.

### 2.4 Population density

For population density, only the mean and approximate 95% confidence intervals (reported as ‘near 20%’ of the mean) for each species and depth class for 1964 were reported. To compare population densities we therefore examined 95% confidence intervals (estimated as mean ± 20% for 1964 data) paired across 1964 and 2020 data. Nonoverlapping 95% confidence intervals indicate a statistically significant difference between samples (*p* < 0.05) (Austin & Hux, 2002). This approach may lead to failure to detect statistically significant differences in cases where confidence intervals do overlap (Schenker & Gentleman, 2001), but we were unable to exclude this possibility using the available data and our conclusions may therefore be overly conservative.

### 2.5 Growth curves

Growth patterns for each species were analysed independently. Growth curves were fitted to the annulus length dataset using the von Bertalanffy growth function (VBGF) (von Bertalanffy, 1938), which has been shown to reflect bivalve growth patterns (Hastie et al., 2000) (Equation 1).

(1) $$L_{t}=L_{\infty }\left(1-e^{-K\left(t-t_{0}\right)}\right)$$

where *t* = age in years, *L*_*t*_ = length at age, and *L*_∞_, *K* and *t*_0_ are constants: *L*_∞_ represents asymptotic length, *K* is the rate at which asymptotic length is attained and *t*_0_ relates to initial size (specifically, it is age at which length is zero).

Growth curve fitting was conducted by nonlinear least squares parameter estimation using the *nls* function in core R, and confidence intervals estimated by bootstrapping using the package car v3.0‐10 (Fox & Weisberg, 2019).

We tested the difference in growth curves between 1964 and 2020 populations using likelihood ratio tests (Kimura, 1980). Since only mean lengths‐at‐age were available for the 1964 dataset, we compared these against means, rather than individual values, for 2020. Assuming constant variance among mean lengths‐at‐age, we constructed a general VBGF in which all three coefficients, *L*_∞_, *K* and *t*_0_, differed between time period, and a set of submodels in which one or more coefficients was constrained (Equations (2.1), (2.2), (2.3), (2.4), (2.5)) following the methodology of Kimura (1980) and Nelson (2019). Each model corresponded to a hypothesis about the equality of VBGF coefficients between time periods. We tested each hypothesis by comparing the submodel against the general model using the chi‐square test, where a value of *p* < 0.05 indicated that the submodel with constrained coefficients fit the data significantly less well than the general model.

(2.1) $$L\sim L_{\infty ,y}\left(1-e^{-K_{y}\left(t-t_{0,y}\right)}\right)$$

*H*_0_: all parameters vary between time periods.

(2.2) $$L\sim L_{\infty }\left(1-e^{-K_{y}\left(t-t_{0,y}\right)}\right)$$

*H*_1_: *L*_∞,1964_ = *L*_∞,2020_

(2.3) $$L\sim L_{\infty ,y}\left(1-e^{-K\left(t-t_{0,y}\right)}\right)$$

*H*_2:_ *K*_1964_ = *K*_2020_

(2.4) $$L\sim L_{\infty ,y}\left(1-e^{-K_{y}\left(t-t_{0}\right)}\right)$$

*H*_3_: *t*_0,1964_ = *t*_0,2020_

(2.5) $$L_{t}=L_{\infty }\left(1-e^{-K\left(t-t_{0}\right)}\right)$$

*H*_4_: *L*_∞1964_ = *L*_∞2020_, *K*_1964_ = *K*_2020_, *t*_0,1964_ = *t*_0,2020_

### 2.6 Productivity

We estimated total productivity following as closely as possible the method described by Negus (1966). To estimate biomass, we constructed log–log regressions of total wet mass against length and shell wet mass against length (Equation 3), fitting a separate model to data for each species, following Atkinson et al. (2020). Estimates for the coefficients *α* and *β* can be found in Table S1.

(3) $$\log\left(\text{mass}\right)=\alpha +\beta \log\left(\text{length}\right)$$

From this we interpolated estimates for total and shell wet mass for each sampled individual. Total estimated biomass from samples from each depth class was scaled by the area sampled and the proportional area of each depth class within the reach (Table S2) to obtain an estimate of standing biomass density in the river for each species. For comparison with results from Negus (1966), where standing biomass was reported for biomass excluding shells, we reported values for shell mass subtracted from total mass.

To estimate annual productivity, we used the fitted von Bertalanffy models (Equation 1; see Results) to interpolate estimates of length at ages *t* and *t +* 1 for each species. These estimates were passed to the length–mass models (Equation 3; Table S1) to estimate total and shell mass at ages *t* and *t* + 1. We scaled these data by the proportions of each age class and species and by the proportion of each depth class within the sampled area of the reach (Table S2), to obtain estimates of standing biomass density (kg ha^−1^) in years *y* and *y* + 1. Annual productivity (kg ha^−1^ y^−1^) was calculated using Equation 4:

(4) $${\text{Productivity}}_{y}={\text{Biomass}}_{y+1}-{\text{Biomass}}_{y}$$

## 3 RESULTS

### 3.1 Species density and community composition

**FIGURE 1:**
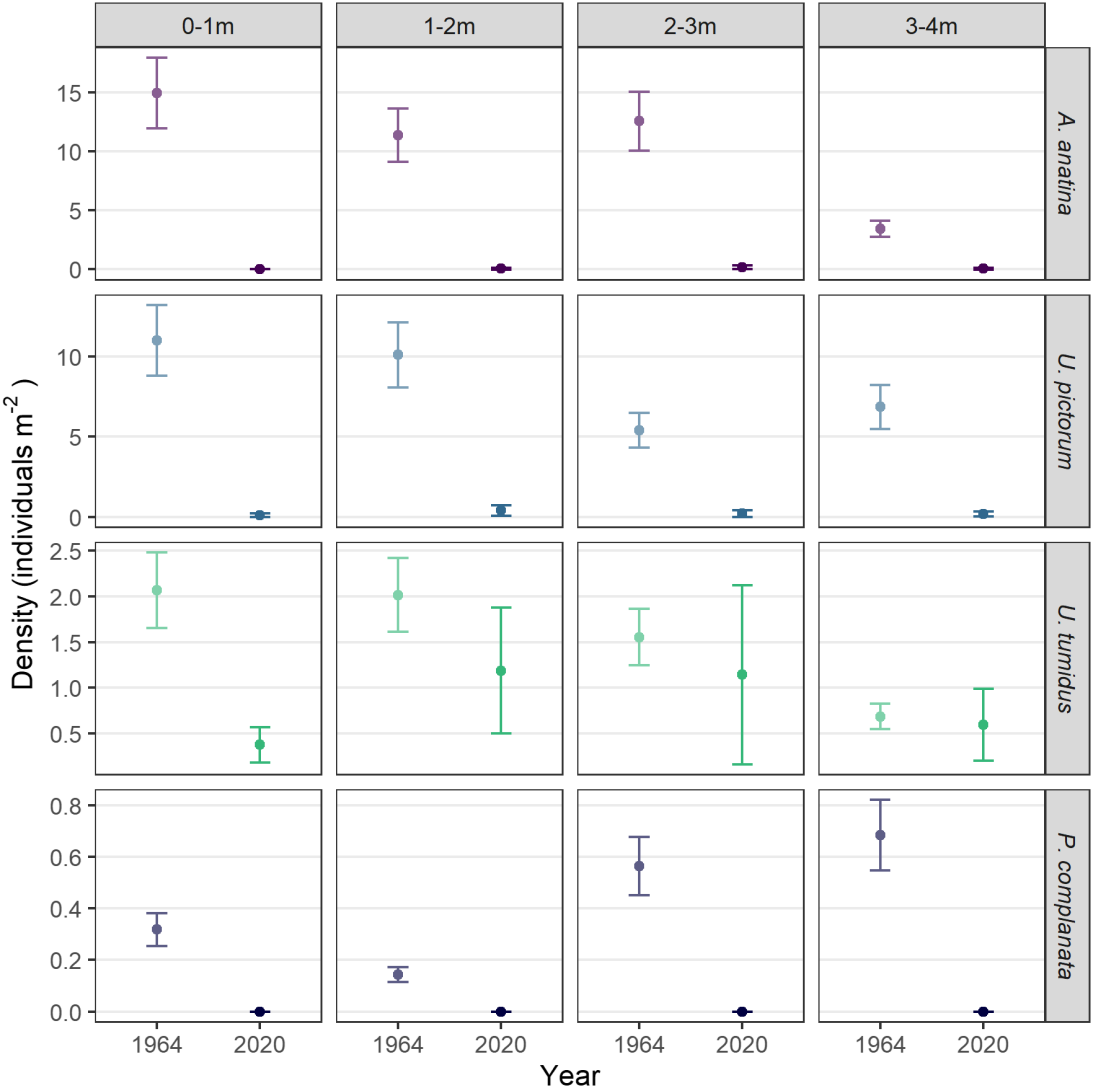
Population densities (individuals m^−2^) of unionid species across depth zones, for 1964 and 2020, with error bars denoting 95% confidence intervals for each species. Nonoverlapping error bars for all cases except *U. tumidus* beyond 1 m depth indicate statistically significant differences between 1964 and 2020, *p* < 0.05. N.B. for ease of within‐plot comparison, *y*‐axis scales are different for each species.

Between 1964 and 2020, unionid density in the River Thames at Reading declined to 6.34% of 1964 levels (Figure 1). Nonoverlapping 95% confidence intervals indicated statistically significant declines (*p* < 0.05) in population density for *A. anatina* and *U. pictorum* in all depth classes. *A. anatina* fell from a mean density of 11.8 individuals m^−2^, aggregated across depths, in 1964 to 0.13 individuals m^−2^ in 2020; and *U. pictorum* fell from 6.8 individuals m^−2^ in 1964 to 0.2 individuals m^−2^ in 2020. In contrast, for *U. tumidus* confidence intervals were only nonoverlapping in the 0–1 m depth zone, and we could not infer statistically significant differences in density at other depths; mean density was 1.6 individuals m^−2^ in 1964 and 1.0 individuals m^−2^ in 2020. No live *Pseudanodonta complanata* (called *Anodonta minima* by Negus) were located during sampling in 2020, down from a sampled density of 0.5 individuals m^−2^ in 1964, although a total of 17 shells occurred in samples.

**FIGURE 2:**
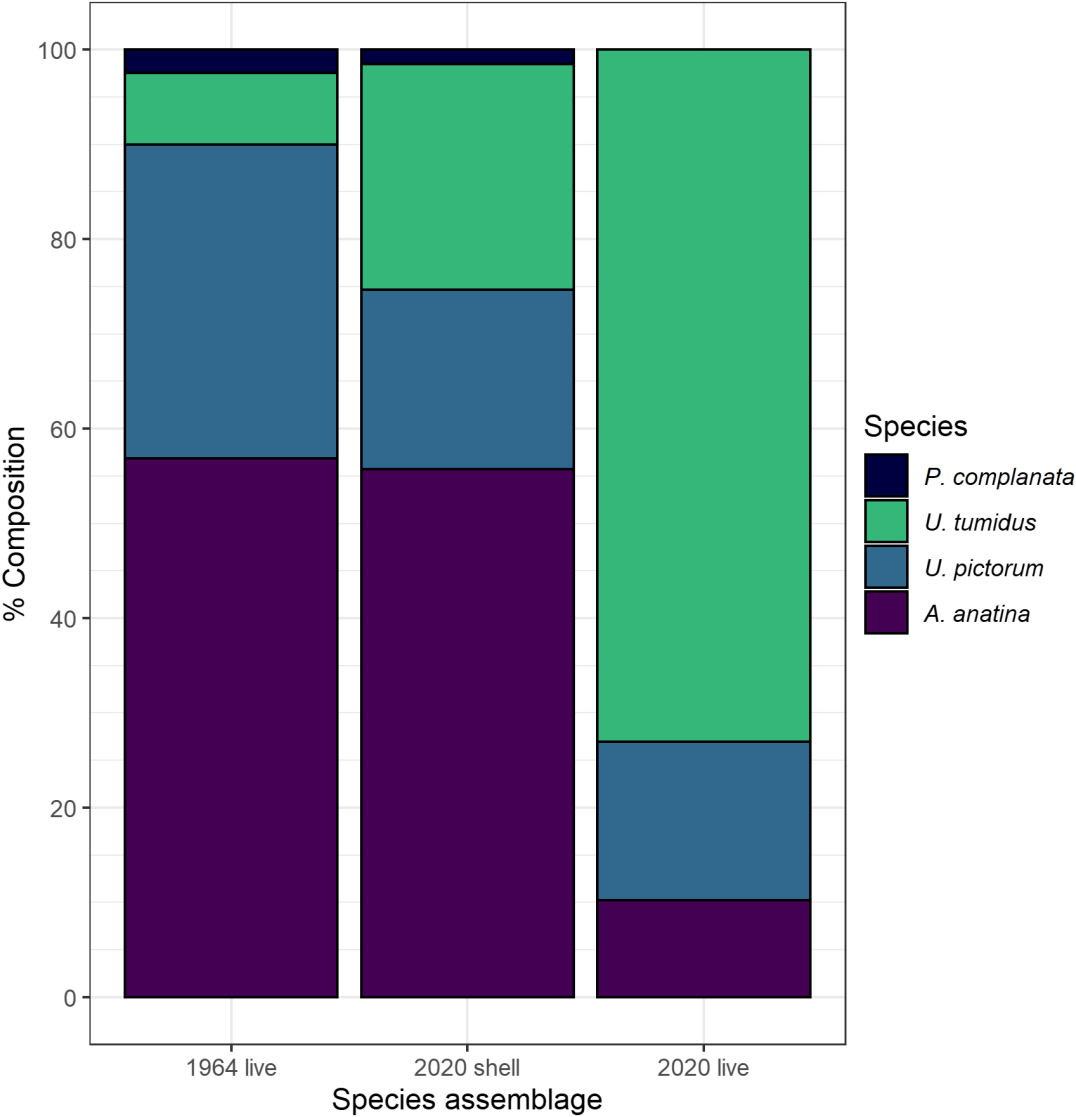
Relative abundance as proportion of total unionid population density of unionid species: 1964 live individuals, 2020 shells (*n* = 1138 shells) and 2020 live individuals (*n* = 225 individuals) No information on number of samples for 1964 is available. Densities are aggregated for all depth classes, using weightings by relative area of each depth zone in the reach.

These differing degrees of decline have resulted in a switch in species dominance within unionid communities (Figure 2). *U. tumidus* has increased as a proportion of overall unionid frequency from 7.6% in 1964 to 73.0% in 2020, while *A. anatina*, *U. pictorum* and *P. complanata* have all decreased (*A. anatina*, 56.9% to 10.2%; *U. pictorum*, 33.1% to 16.7%; *P. complanata*, 2.4% to 0%). Shells collected in 2020 showed a greater similarity to live community composition in 1964, with a greater relative frequency of *A. anatina* and *P. complanata* (55.7% and 1.5% respectively).

No nonnative bivalves were found at the site during 1964 surveys (Negus, 1966; C. Negus, pers. comm.). During 2020 surveys both the zebra mussel *Dreissena polymorpha* (196 live individuals across all samples) and the Asian clam *Corbicula fluminea* (three live individuals) were collected.

We also found a shift in population densities across depth classes (Figure 1). In 1964 total unionid density decreased with increasing depth, with density highest in the shallowest (0–1 m) areas and lowest in the deepest (3–4 m) areas. In contrast, in 2020 density was highest in the intermediate depth zones (1–2 m and 2–3 m), driven by density of *U. tumidus*, although declines were observed across all depth classes.

### 3.2 Age structure

**FIGURE 3:**
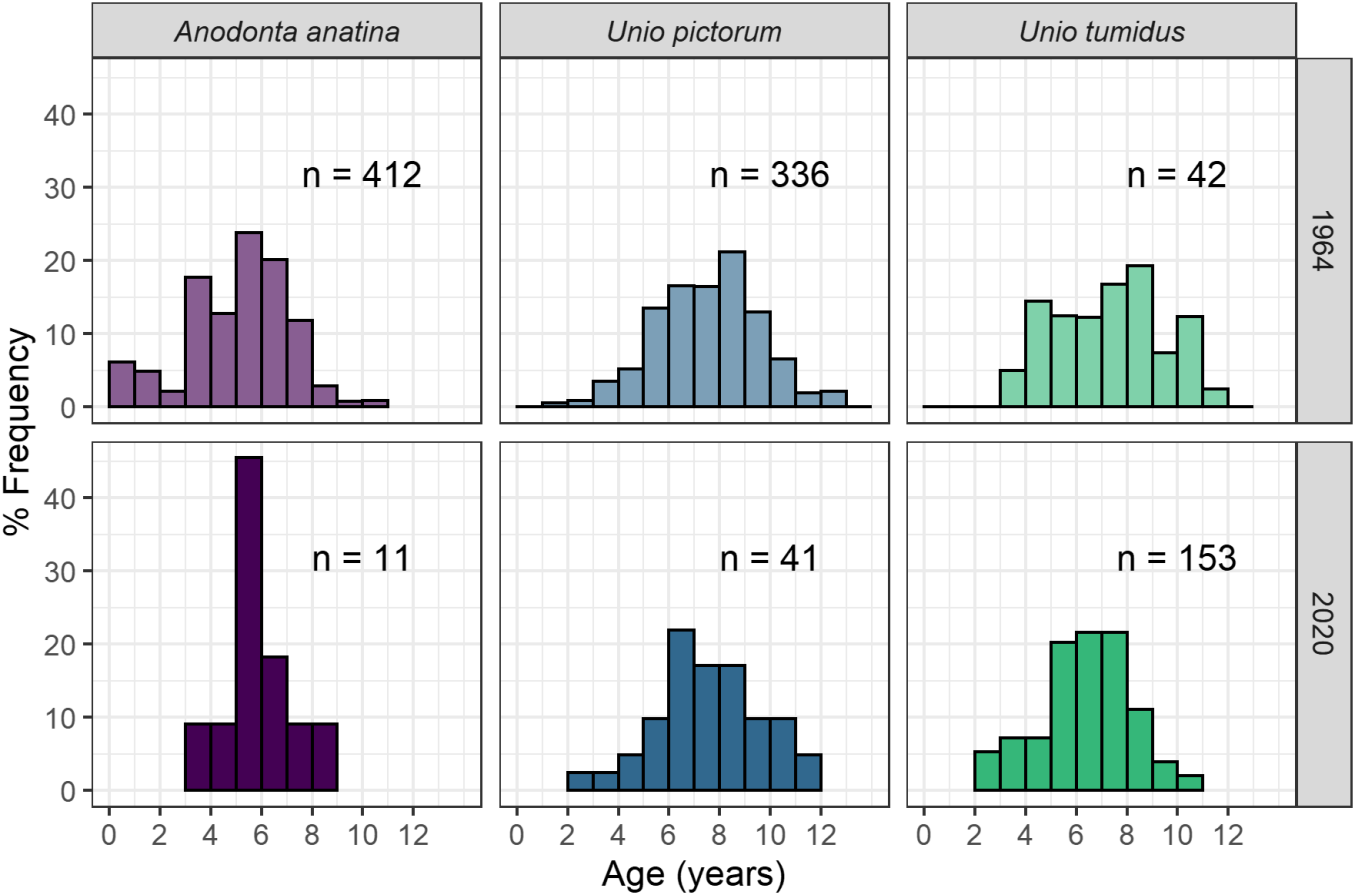
Species‐specific age frequency distributions, compared across 1964 and 2020 surveys. For comparison, values are plotted as % frequency since marked differences in absolute population size to 2020 makes a comparison based on these values difficult to visualise. Age differed significantly between years for *U. tumidus* but not *A. anatina* or *U. pictorum*.

We found no significant differences in age distribution using Fisher's exact test for either *A. anatina* (*p* = 0.771) or *U. pictorum* (*p* = 0.809) (Figure 3) between 1964 and 2020. Age distribution of *U. tumidus* differed significantly between 1964 and 2020 (*p* = 0.016), although this difference was relatively small (mean age in 1964 = 6.8; mean age in 2020 = 5.9) and unlikely to reflect major changes in population age structure.

### 3.3 Growth curves

**FIGURE 4:**
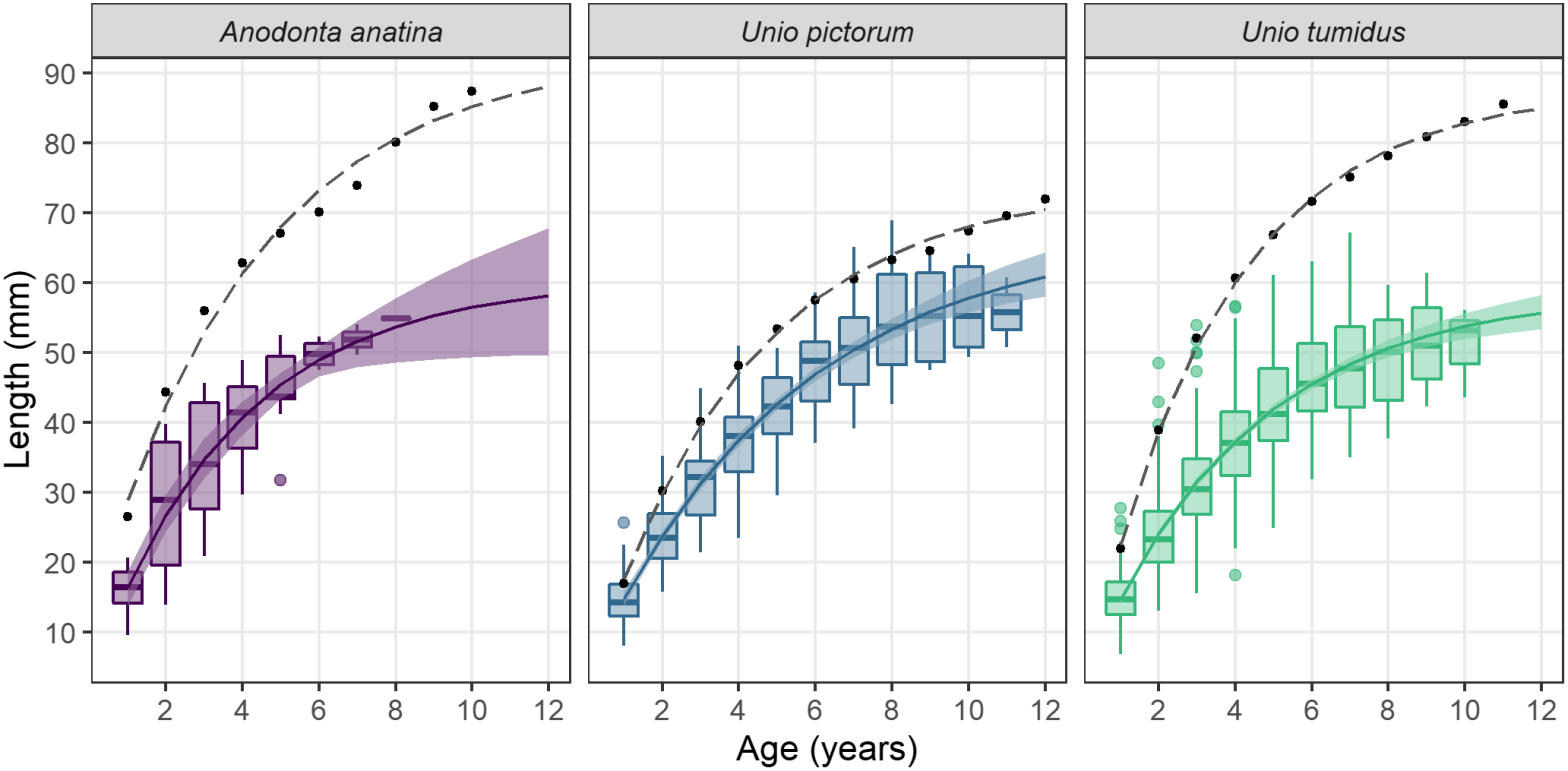
Average length at age of mussels in 1964 (black, dashed line) and in 2020 (shaded, solid line). Boxplots were not plotted for 1964 data as only data on mean lengths for each age were available. Fitted von Bertalanffy growth curves are shown for 1964 (dashed line) and 2020 (solid line), with associated bootstrapped 95% confidence intervals for 2020. von Bertalanffy growth parameters *L*_∞_, *K* and *t*_0_ fitted separately for each time period are shown in Table 1.

Von Bertalanffy growth functions (VBGFs) fitted to species length‐at‐age data for 1964 and 2020 (Figure 4; Table 1) showed a decrease in length at age across all ages in 2020, a pattern consistent across all three species surveyed. For all species and all ages, mean values for length at age in 1964 fell well above the upper quartiles for equivalent 2020 data, and fitted VBGFs for 1964 did not overlap with bootstrapped 95% confidence intervals for VBGFs for 2020 (Figure 4).

**TABLE 1:** von Bertalanffy parameters fitted separately to each species and time period using nonlinear least squares regression, with 95% confidence intervals. Values for 2020 are fitted to our raw dataset, and values for 1964 are fitted to mean length at age. Residual standard errors (RSEs) with degrees of freedom (subscript, parentheses) are provided for each model

Species	Year	*L*_∞_	*K*	*t*_0_	RSE_(df)_
*A. anatina*	1964	92.9 ± 11.6	0.236 ± 0.103	−0.581 ± 0.766	2.72_(7)_
	2020	60.4 ± 17.5	0.270 ± 0.195	−0.177 ± 0.711	6.67_(56)_
*U. pictorum*	1964	74.3 ± 2.1	0.245 ± 0.027	−0.112 ± 0.207	0.97_(10)_
	2020	67.6 ± 7.0	0.187 ± 0.045	−0.324 ± 0.299	5.65_(287)_
*U. tumidus*	1964	87.8 ± 2.1	0.286 ± 0.028	−0.037 ± 0.154	0.87_(8)_
	2020	58.9 ± 4.1	0.239 ± 0.041	−0.205 ± 0.186	6.51_(894)_

The VBGF coefficient *L*_∞_ differed significantly between time periods for all three species (Table 2). In contrast, the *K* and *t*_0_ coefficients did not differ significantly between time periods in any species. AIC comparison further showed the lowest values for models which allowed *L*_∞_ to differ between time periods (Table S3).

**TABLE 2:** Likelihood ratio tests comparing von Bertalanffy growth parameters between time periods. Reject hypothesis of equality of parameters when *p* < 0.05

Species	Model comparison	Hypothesis	*χ* ^2^_(df)_	*p*
*A. anatina*	*H*_0_ – *H*_1_	*L*_∞1964_ = *L*_∞2020_	6.31_(1)_	**0.012**
	*H*_0_ – *H*_2_	*K*_1964_ = *K*_2020_	0.05_(1)_	0.823
	*H*_0_ – *H*_3_	*t*_0,1964_ = *t*_0,2020_	0.87_(1)_	0.351
	*H*_0_ – *H*_4_	*L*_∞1964_ = *L*_∞2020_, *K*_1964_ = *K*_2020_, *t*_0,1964_ = *t*_0,2020_	64.54_(3)_	**<0.001**
*U. pictorum*	*H*_0_ – *H*_1_	*L*_∞1964_ = *L*_∞2020_	16.17_(1)_	**<0.001**
	*H*_0_ – *H*_2_	*K*_1964_ = *K*_2020_	1.48_(1)_	0.224
	*H*_0_ – *H*_3_	*t*_0,1964_ = *t*_0,2020_	0.23_(1)_	0.632
	*H*_0_ – *H*_4_	*L*_∞1964_ = *L*_∞2020_, *K*_1964_ = *K*_2020_, *t*_0,1964_ = *t*_0,2020_	76.54_(3)_	**<0.001**
*U. tumidus*	*H*_0_ – *H*_1_	*L*_∞1964_ = *L*_∞2020_	42.80_(1)_	**<0.001**
	*H*_0_ – *H*_2_	*K*_1964_ = *K*_2020_	0.11_(1)_	0.740
	*H*_0_ – *H*_3_	*t*_0,1964_ = *t*_0,2020_	0.16_(1)_	0.693
	*H*_0_ – *H*_4_	*L*_∞1964_ = *L*_∞2020_, *K*_1964_ = *K*_2020_, *t*_0,1964_ = *t*_0,2020_	118.95_(3)_	**<0.001**

### 3.4 Biomass and production

As a result of declines in both population density and growth rate, estimated biomass and annual production by unionids decreased between 1964 and 2020 (Table 3). This decrease was greatest for *A. anatina*, where production was nearly 80 times lower in 2020 than 1964, while in *U. pictorum* production was over 20 times lower. In contrast, *U. tumidus* productivity fell to only around half that of 1964. Across all species, total estimated biomass production was over 13 times lower in 2020 than 1964.

**TABLE 3:** Estimated biomass and annual production (kg ha^−1^ y^−1^) for each species, calculated as a weighted average across all age and depth classes. Values for 1964 are those reported by Negus (1966)

Species	Biomass (kg ha^−1^) 1964	Biomass (kg ha^−1^) 2020	Productivity (kg ha^−1^ y^−1^) 1964	Productivity (kg ha^−1^ y^−1^) 2020
*Unio pictorum*	382.1	18.5	52.9	2.58
*Unio tumidus*	156.1	73.0	20.0	11.1
*Anodonta anatina*	648.2	5.75	132.2	1.72
*Pseudanodonta complanata*	20.9	0.0	Not reported	0.0
Total	1207.3	97.2	205.1	15.4

### 3.5 Water quality

**FIGURE 5:**
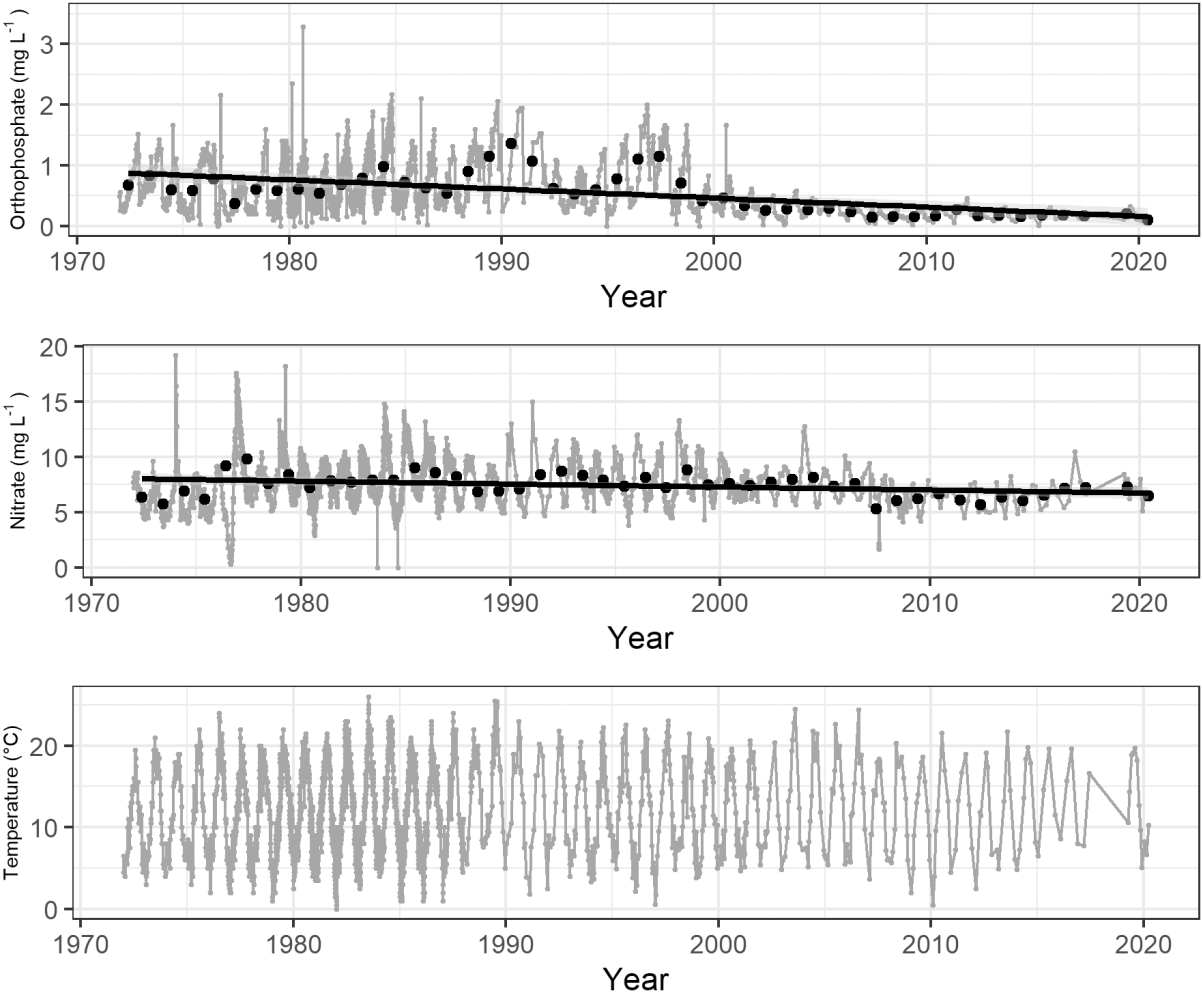
Water quality time series for the Thames at Caversham Weir showing orthophosphate and nitrate concentrations (mg L^−1^) and water temperature (°C). Individual measurements are plotted in grey, with mean annual concentrations in black. Linear trends fitted to annual means are shown in black. One extreme value for orthophosphate was excluded for plotting (15 mg L^−1^ on 5 October 1984). Annual mean concentration for both orthophosphate and nitrate declined significantly with time (orthophosphate: *R* ^2^adj = 0.409; nitrate: *R* ^2^adj = 0.125).

Annual mean concentrations of orthophosphate declined significantly over time (*F*_1,46_ = 33.6, *p* < 0.001; Figure 5; Table S4), with a noticeable drop in concentration and fluctuation over the course of each year around 1999–2000, coinciding with the introduction of stronger sewage treatment legislation.

Annual mean nitrate concentration also showed a significant decline over time (*F*_1,46_ = 7.69, *p* = 0.008; Figure 5; Table S4), although the size of this decline was much less marked.

There were no significant changes in mean, maximum or minimum water temperature over the period (Figure 5).

## 4 DISCUSSION

Few historical studies of freshwater mussels have collected detailed quantitative data on spatial patterns of species composition, population density and growth rates; even fewer have assessed production. One such study, conducted in the River Thames at Reading (Negus, 1966), has provided a unique opportunity to produce a quantitative assessment of unionid population trajectories in a major river over the past 50 years. We found that unionid populations in the River Thames at Reading have declined alarmingly since they were surveyed in 1964. In addition, all species show markedly reduced size at age and reach smaller size compared to 1964 populations. Decreases in both per‐individual size and population density have led to declines in productivity across all species, with total annual productivity in 2020 reduced to 7.5% of 1964 levels. This is likely to have resulted in part from environmental shifts in the ecosystem: concentrations of nitrogen and phosphorus have decreased, while the invasive bivalves *Dreissena polymorpha* and *Corbicula fluminea* are now present at the site. This study is limited by the availability of historical data to a single site, which necessarily limits the conclusions that can be drawn about the drivers of decline. Comparisons with similar studies elsewhere in Europe can help to form a broader picture.

Population densities decreased significantly across all species and depth zones, except in *U. tumidus* where the decrease was statistically significant only in the 0–1 m depth zone. This overall pattern of cross‐species decline reflects wider trends of decline in unionid populations globally (Lopes‐Lima et al., 2018). Studies elsewhere in Europe have also found that *U. tumidus* populations have declined less than other sympatric unionid species (Arter, 1989; Lewandowski & Kołodziejczyk, 2014; Ożgo et al., 2021), and this may suggest a greater tolerance for environmental stressors by this species. Conversely, it is likely that *P. complanata* has been extirpated at the site because although shells of the species were present, we found no live individuals. The species is listed as Near Threatened by both the European (Cuttelod et al., 2011) and Great Britain (Seddon et al., 2014) Red Lists and is a Priority Species under the UK Biodiversity Action Plan (BRIG, 2007). Studies elsewhere in Europe have shown that *P. complanata* can be more strongly affected than other unionids by environmental stressors (Ćmiel et al., 2019) and invasive zebra mussels (Ożgo et al., 2020), and may be among the first species to be extirpated from communities (Lewandowski & Kołodziejczyk, 2014).

Population declines in freshwater mussels are often associated with a reduced recruitment rate, leading to an aging population and right‐skewed age distribution (e.g. Hastie & Toy, 2008). In our study, age‐frequency distributions were broadly similar in 1964 and 2020, and only *U. tumidus* showed a significant (but small) change in mean age, from 6.8 to 5.9 years. This could suggest that, despite lower recruitment than in 1964, populations may have reached a new stable state. In addition, the relatively low number of individuals in younger age classes in both the 1964 and 2020 samples may reflect under‐sampling of younger and smaller mussels which were less likely to be captured by dredging or hand‐sampling.

The appearance of two nonnative bivalves (*D. polymorpha* and *C. fluminea*) may have contributed to population declines, although densities, especially for *C. fluminea*, appeared relatively low compared to those recorded elsewhere. Neither species was recorded in the 1964 study (confirmed by C. Negus, pers. comm.). At high densities, *D. polymorpha* infestation is linked to declines in native unionids (Nalepa, 1994; Sousa et al., 2011) due to competition and biofouling (Sousa et al., 2011; Strayer & Malcom, 2018). Among European unionid species, *A. anatina* exhibits a greater reduction in tissue mass in infested versus uninfested individuals than *U. pictorum* (Sousa et al., 2011), a pattern which may generalise to larger‐ and thinner‐shelled *Anodontinae* compared with more robust *Unioninae* (Nalepa, 1994). This may partially explain the much greater reduction in population density of *A. anatina* in our surveys compared with *Unio* species, and especially *U. tumidus*. Similar effects have been found in Lake Hallwil in Switzerland (Arter, 1989), where mussel communities surveyed in 1915 and later in 1986 shifted from *Anodonta* to *Unio* dominance, associated with invasion by *Dreissena*. In Lake Mikolajskie in Poland, Lewandowski and Kołodziejczyk (2014) also reported a greater decline in *Anodonta* species relative to *U. tumidus* between 1972 and 2008, in a community with heavy biofouling by *Dreissena*. *Dreissena* can also have strong impacts on *Pseudanodonta complanata*, causing shell deformation and impairing burrowing and anchoring (Ożgo et al., 2020). In contrast, in the River Ognon in France where the dominant invasive is *Corbicula fluminea*, which does not cause biofouling, unionid declines from 1977 to 2007 were more uniform across species (Mouthon & Daufresne, 2010): impacts here are probably due to competition with *Corbicula* for food rather than biofouling, which may be equally deleterious across all the unionid mussel species. Although *Corbicula fluminea* was also found at the Thames study site, density is probably currently too low to affect unionid populations significantly. It is noteworthy that colonisation by invasive bivalves does not always lead directly to declines in unionid populations: in the River Szeszupa in Poland, Ożgo et al. (2021) did not find significant impacts of *Dreissena* on unionid populations, although this was explained by highly intact habitats.

Other biotic interactions are also important in unionid population dynamics, including interactions with fish hosts by mussel larvae (glochidia), and declines in populations of host fish can have knock‐on impacts on unionids (Modesto et al., 2018). In the Thames, *Gasterosteus aculeatus* and *Perca fluviatilis* were the most important host species for unionids (Berrie & Boize, 1985), carrying respectively 55.6% and 33.7% of all sampled glochidia. Thames fish populations appear and are expected to remain stable (Hughes & Willis, 2000), with *G. aculeatus* comprising 44.7% of total fish sampled (Araujo et al., 1999). Unionid populations in the Thames are therefore unlikely to be significantly threatened by loss of fish hosts. Furthermore, the unionid species in the River Thames are host generalists, which should provide some buffering against declines in particular species.

We found reduced growth in 2020 compared to 1964 across all species. Unionid populations have highly variable growth patterns even within species, with von Bertalanffy growth parameters differing both within and between locations (Haag & Rypel, 2011; Muller et al., 2021). Variations in size and shell morphology are correlated with environmental variables including flow regime (Zieritz & Aldridge, 2009), mortality rate and waterbody trophic status (Muller et al., 2021). Flow rate is unlikely to have contributed significantly here since analysis has shown no significant changes in maximum flow or flood events in the period since 1951 for the River Thames (Marsh & Harvey, 2012). However, we did find evidence for changing nutrient levels at the site, with decreases in both nitrate and, most strongly, in phosphate concentration shown by long‐term monitoring data from close to the study site. This is probably largely due to increased regulation of wastewater effluents and the establishment and improvement of sewage treatment works (Kinniburgh & Barnett, 2009; Neal, Jarvie, et al., 2010; Neal, Martin, et al., 2010). Consequently, chlorophyll *a* concentration, a proxy for phytoplankton density, has also decreased in the River Thames in recent years (Bowes et al., 2012; Kinniburgh & Barnett, 2009).

The impact of changing nutrient level on mussel populations is complex and is dependent on the starting nutrient concentration. Nutrient reduction in nutrient‐rich waters may benefit mussels by limiting algal blooms and the development of hypoxic conditions as algae decomposes. Highly eutrophic conditions can be harmful to mussels: in the River Jorka in Poland, nutrient enrichment may have contributed to the extirpation of some unionid species at sites experiencing periods of anoxia (Kołodziejczyk et al., 2009), and in a study of Estonian lakes, mussel density reductions were linked to eutrophication‐driven hypoxia (Timm et al., 2006). Conversely, nutrient reduction in nutrient‐poor waters may be harmful to mussels since reduced algal growth may start to limit food availability. Reduced nutrient availability may therefore have led to a bottom‐up reduction in secondary production in mussel populations, with lower individual growth rates and possibly lower carrying capacity. Nutritional stress may also have increased susceptibility to other stressors including *Dreissena* invasion.

Changing trophic status has been shown to affect individual growth rates in mussels at other sites. In Lake Hallwil in Switzerland, *U. tumidus* in 1986 grew at a higher rate than individuals collected in 1915 (Arter, 1989), a shift attributed to the lake's transition from mesotrophic to eutrophic, with greater food availability supporting faster growth. Similar trends have also been shown over much longer time‐scales: in the Illinois River, USA, a study comparing archaeological and present‐day mussels found an increase in growth rates over the thousand‐year period to 2013, with most of this increase occurring since 1897 (Fritts et al., 2017). This coincides with enriched δ^15^N and δ^13^C shell isotopic signatures, indicating increased nutrient levels. The reverse trend—a decrease in nutrient enrichment leading to reduced growth rates—may therefore have occurred in the Thames.

In another study comparing historical and current growth rates in unionids, Czerniejewski et al. (2021) also found a decrease in growth rates in modern‐day mussels from the Oder Estuary in Poland when compared with shells from a medieval midden from the same location. Here, however, the authors suggest that climate rather than nutrient availability is the most likely cause, with favourable temperatures during the Medieval Climatic Optimum leading to elevated growth. We found no evidence that mean annual temperature at our study site has changed since 1970, and decreasing nutrient concentrations provide a more likely explanation.

Both Arter (1989) and Czerniejewski et al. (2021) found that increases in growth rate were associated with shorter life span, since patterns of resource allocation differ according to life history. In contrast, in a study of mussel populations in Polish lakes of differing trophic status, those in more eutrophic environments showed a reverse effect: higher mortality, potentially resulting from anoxia, led to earlier maturation at a lower maximum size (Muller et al., 2021). We found no evidence that reduced growth rate in the Thames mussel population has been associated with either an increase or decrease in life span, since age structure between 1964 and 2020 has remained similar.

Since this study is necessarily limited to a single site, we cannot assess how widespread the reduction in species density and production may be throughout the river. In particular, it is clear that mussel population trajectories can vary greatly, even within the same broad ecosystem (Kołodziejczyk et al., 2009). We therefore recommend that further assessments of mussel population dynamics, and in particular secondary production, should be conducted across habitats, which could facilitate a broader meta‐analysis to assess how widely applicable these patterns are in both space and time.

Although decreases in growth rate and population density may typically be seen as warning signs that a population is under threat, the individual growth rate reductions we found in our study population may instead be interpreted as a reversion to preanthropogenic levels. Data from prehistoric mussel populations have shown that over millennial time‐scales individual body size in some populations has increased, attributed to anthropogenic nutrient enrichment (Fritts et al., 2017). The historical 1964 data used in the present study do not represent a pristine ‘baseline’ by any means, but an ecosystem that was already highly anthropogenically enriched. In this context, declines in growth rate (although not density), probably resulting in part from nutrient reductions, may actually represent a return to a more ‘natural’ state, down from anthropogenically elevated levels which may have existed in the 1960s.

This study demonstrates the vital importance of maintaining up‐to‐date knowledge about wild populations, including for species not currently classed as threatened. Our results suggest that declines in nonprotected and supposedly common species may be going unnoticed (Ożgo et al., 2021). Such changes are likely to have significant impacts on other freshwater species and broader ecosystem functioning. In particular, we highlight the need to avoid relying on old data on growth patterns, species distributions and population dynamics, which we have shown to change dramatically over time even within one location. Instead, such data should be regarded as a valuable historical snapshot of a population, which should be combined with regular resampling to elucidate patterns of decline, which remain poorly understood in unionids (Lopes‐Lima et al., 2021). Evidence is building to suggest that some mussel declines may result from single catastrophic events, including exposure to novel pathogens (Richard et al., 2020), rather than being linear over a long period. More frequent sampling of populations, yielding higher temporal resolution data, is required to assess the dynamics of these population declines and allow better mapping to specific causes or trigger events.

## AUTHOR CONTRIBUTIONS

Isobel Ollard and David C. Aldridge designed the study and carried out sampling; Isobel Ollard conducted further data collection and analyses and wrote the first draft of the manuscript; Isobel Ollard and David C. Aldridge contributed critically to revisions and gave final approval for publication.

## CONFLICT OF INTEREST

The authors declare no conflict of interest.

## Supporting information


Table S1
Table S2
Table S3
Table S4

## Data Availability

Data are available through the Apollo Digital Repository https://doi.org/10.17863/CAM.8001 (Ollard & Aldridge, 2022).
