# Supplementary material for: Declines in freshwater mussel density, size and productivity in the River Thames over the past half century
Source: J Anim Ecol. 2022 Nov 27;92(1):112–23. doi: 10.1111/1365-2656.13835 (PMC10100129; doi:10.1111/1365-2656.13835)
Supplement: Supplementary file 1 — Table S1 Table S2 Table S3 Table S4 [file JANE-92-112-s001.docx]

Table S1: Log-log regression coefficients and standard errors for mass – length regressions. *** p < 0.001; ** p < 0.01; * p < 0.05

|  | Species | α | β | F_(df)_ | Adjusted R^2^ |
| --- | --- | --- | --- | --- | --- |
| Total wet mass | *A. anatina* | -8.76 ± 4.77 ** | 2.87 ± 1.23 ** | 55.4_(1,3)_ ** | 0.932 |
|  | *U. pictorum* | -7.68 ± 2.48 *** | 2.63 ± 0.62 *** | 89.5_(1,10)_ *** | 0.889 |
|  | *U. tumidus* | -8.94 ± 0.68 *** | 2.99 ± 0.18 *** | 1155_(1,31)_ *** | 0.973 |
| Shell wet mass | *A. anatina* | -13.5 ± 5.60 ** | 3.76 ± 1.44 ** | 69.0_(1,3)_ ** | 0.944 |
|  | *U. pictorum* | -8.06 ± 4.22 ** | 2.53 ± 1.06 *** | 28.5_(1,10)_ *** | 0.714 |
|  | *U. tumidus* | -9.84 ± 1.13 *** | 3.02 ± 0.30 *** | 435.1_(1,31)_ *** | 0.931 |

Table S2: Weightings for depth zones of the sampled area of the reach. Weightings are based on the depth profile provided by Negus (1966).

| Depth zone | Weighting |
| --- | --- |
| 0-1m | 0.16 |
| 1-2m | 0.08 |
| 2-3m | 0.64 |
| 3-4m | 0.12 |

Table S3: AIC values for models fitted for Kimura’s likelihood ratio test (models correspond to those reported in Table 3)

|  | Model AIC | | | | |
| --- | --- | --- | --- | --- | --- |
| Species | H_0_ | H_1_ | H_2_ | H_3_ | H_4_ |
| *A. anatina* | 84.996 | 89.303 | 83.048 | 83.863 | 143.533 |
| *U. pictorum* | 76.529 | 90.702 | 76.010 | 74.759 | 147.071 |
| *U. tumidus* | 60.277 | 101.072 | 58.388 | 58.433 | 173.227 |

Table S4: Regression coefficients and standard errors for nitrogen and orthophosphate concentration over time. *** p < 0.001; ** p < 0.01; * p < 0.05

| Parameter | Intercept | Slope | Adjusted R^2^ |
| --- | --- | --- | --- |
| Nitrogen | 8.08 ± 0.284 *** | -0.0000733 ± 0.0000264 ** | 0.125 |
| Orthophosphate | 0.914 ± 0.0762 *** | -0.0000410 ± 0.00000708 *** | 0.409 |
